# Familiarization with meaningless sound patterns facilitates learning to detect those patterns among distracters

**DOI:** 10.3389/fpsyg.2022.957389

**Published:** 2022-09-14

**Authors:** Matthew G. Wisniewski

**Affiliations:** Department of Psychological Sciences, Kansas State University, Manhattan, KS, United States

**Keywords:** frozen noise, perceptual learning, temporal dynamics, pattern detection, learning rate, informational masking, auditory memory

## Abstract

Initially “meaningless” and randomly generated sounds can be learned over exposure. This is demonstrated by studies where repetitions of randomly determined sound patterns are detected better if they are the same sounds presented on previous trials than if they are novel. This experiment posed two novel questions about this learning. First, does familiarization with a sound outside of the repetition detection context facilitate later performance? Second, does familiarization enhance performance when repeats are interleaved with distracters? Listeners were first trained to categorize a unique pattern of synchronous complex tone trains (210 ms in duration) from other tone trains with similar qualities (familiarization phase). They were then tasked to detect repeated pattern presentations interleaved with similar distracters in 4.2 s long excerpts (repetition detection phase). The familiarized pattern (Familiar Fixed – FF), an unfamiliar pattern that remained fixed throughout (Unfamiliar Fixed – UF), or patterns that were uniquely determined on each trial (Unfamiliar Unfixed – UU) could be presented as repeats. FF patterns were learned at a faster rate and achieved higher repetition detection sensitivity than UF and UU patterns. Similarly, FF patterns also showed steeper learning slopes in their response times (RTs) than UF patterns. The data show that familiarity with a “meaningless” sound pattern on its own (i.e., without repetition) can facilitate repetition detection even in the presence of distracters. Familiarity effects become most apparent in the potential for learning.

## Introduction

Sounds that are familiar to us can show advantages in perceptual processing compared to unfamiliar sounds. This is a phenomenon indicative of *perceptual learning* (for review, see Wright and Zhang, 2009; Irvine, 2018; Maniglia and Seitz, 2018). The impacts of sound familiarity can appear rather quickly with initially “meaningless” sound stimuli. For instance, Agus et al. (2010) found that repetitions of random Gaussian noise samples were detected with greater sensitivity if they were the same noise samples presented on previous trials than if they were unfamiliar samples generated under the same constraints (also, see Agus and Pressnitzer, 2013). These frozen noise effects occur after a few trials (Agus et al., 2010), can be seen for noise samples as short as 10 ms (Andrillon et al., 2015), and are observable a month or more after initial exposure (Viswanathan et al., 2016; Bianco et al., 2020; Agus and Pressnitzer, 2021). Also notable is that they can occur with randomly generated tone pattern stimuli that closely resemble the acoustic characteristics of many real-world sounds and receptive field properties of auditory cortical neurons (DeCharms et al., 1998; Sohoglu and Chait, 2016; Bianco et al., 2020; Herrmann et al., 2021). This introduces exciting possibilities for studies of perceptual learning with complex sounds that are uncorrupted by previous knowledge on the part of listeners (as is the case with speech or environmental sounds).

In these previous works, sound repetitions mostly occurred consecutively with no intervening stimuli. In real-world environments, however, repetitions are rarely experienced without distracters. Examples include environmental noise during alarm sound repetition (Edworthy et al., 2018) and accompaniment to the melody of a single musical instrument (Waggoner, 2011). It is well known that detection can be hindered in these types of scenarios (Durlach, 2006). In one recent study, it was found that listeners could learn repeated tone patterns interleaved with other random patterns over the course of trials, but this learning was limited compared to patterns presented without distracters (Bianco et al., 2020). Also, this learning occurred within the repetition task itself. This leaves ambiguity as to whether learning entails memory for a specific sound pattern, or a learned strategy to listen for the sound quality that results from pattern repeats (e.g., “wooshing”; Warren and Bashford, 1981). Distinguishing these will be informative for development of perceptual learning models where these possibilities can be associated with different mechanisms (e.g., plasticity in signal representations or changes in top-down selective attention). Whether or not perceptual learning transfers to a listening situation where repeats are interleaved with similar sounds is also undetermined. Such work is needed to assess the predictions of several learning theories that the benefits of perceptual learning lie in the potential for further learning on untrained tasks, not just performance in the trained task (e.g., Gibson, 1969; Mercado III, 2008; Goldstone et al., 2010; Seppänen et al., 2013). This is especially needed in tasks that induce learning with stimuli that do not have preexisting biases associated with them (e.g., speech). The current experiment addresses both of these questions.

In a first familiarization phase, synchronous tone train patterns having randomly generated frequencies between 300 and 1,200 Hz were presented to listeners. Instructions were to answer whether a sound was “Sound A” or “Sound B.” While Sound A was frozen throughout this phase (“Familiar-Fixed” – FF), Sound B was generated randomly on each trial (“Unfamiliar-Unfixed” – UU). In a following repetition detection phase of the experiment, listeners were tasked to detect repeating patterns of synchronous tone trains within a relatively long excerpt (4.2 s in length) containing multiple tonal patterns. Previously familiarized (FF) and unfamiliar patterns (Unfamiliar – Fixed – UF; UU) were presented within excerpts. On “repeating” trials, repeating patterns were always interleaved with other randomly generated tonal patterns. It was hypothesized that familiarization with a “meaningless” sound pattern presented by itself would lead to differences in repetition detection sensitivity, response time, and learning rate between familiar and unfamiliar sounds. It was expected that this effect would be observable when patterns were interleaved with other patterns having similar characteristics.

## Materials and methods

### Listeners

Listeners were 31 individuals enrolled in General Psychology courses at Kansas State University. The *N* was determined *a priori* based on a presumed effect size of Cohen’s *d* = 0.5 for a comparison between FF and UF sounds (achieving >80% power for a single-sided hypothesis). All signed an informed consent document. All procedures were approved by Kansas State University’s institutional review board. All listeners reported normal hearing. One listener was eliminated from analysis for failing to perform above chance in the familiarization phase of the experiment.

### Apparatus

Sounds were presented by an RME UC Fireface device over Sennheiser HD-280 closed-back headphones in a WhisperRoom sound-attenuating booth. Experimental procedures and stimuli were programmed in Matlab. Listeners made responses using a computer mouse to click buttons on an on-screen GUI.

### Stimuli

**Figure 1:**
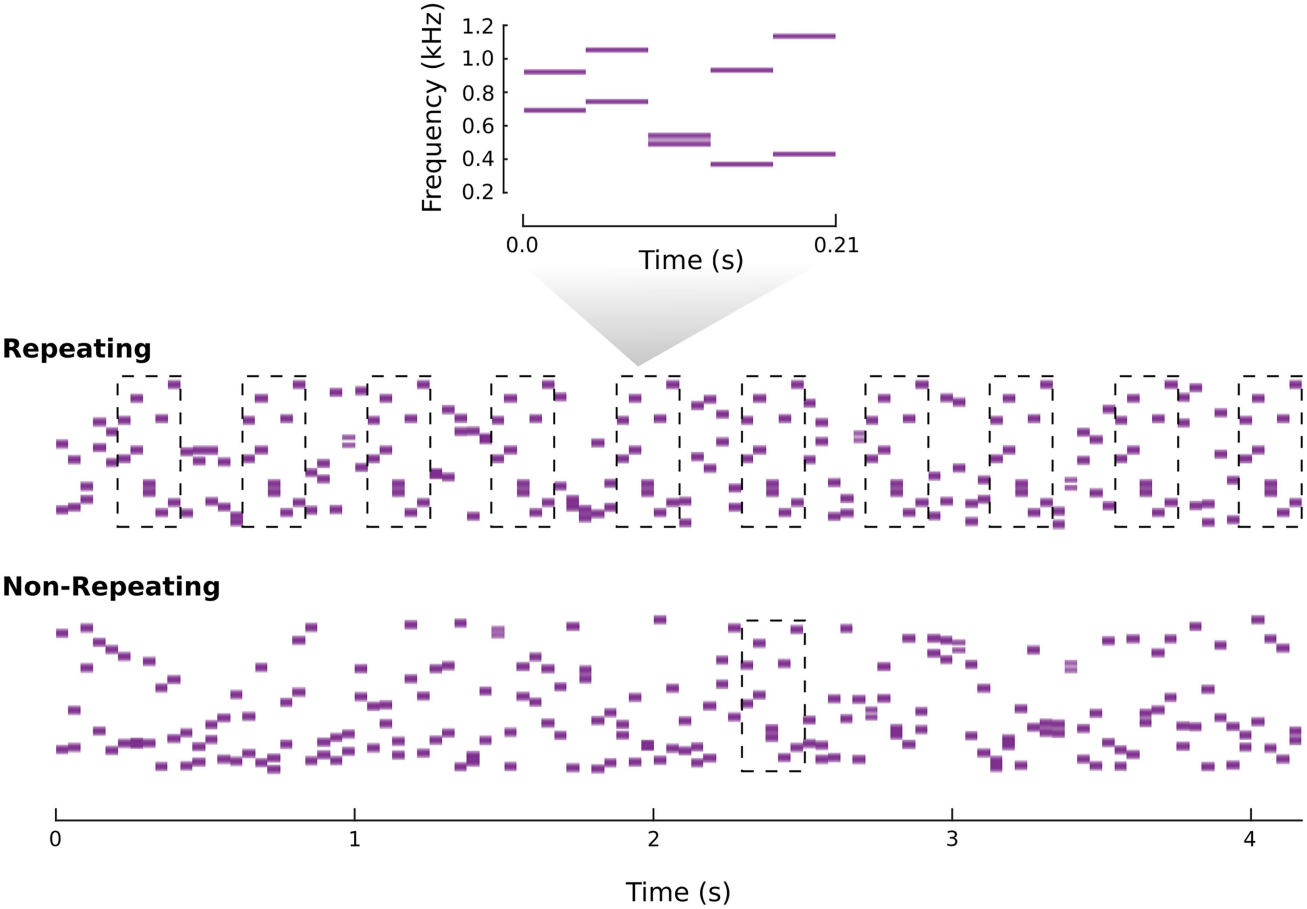
Spectrogram example of repeating and non-repeating excerpts of synchronous tone trains. The breakout spectrogram shows an example pattern made up of 5 randomly generated multitone complexes. The dashed boxes in the repeating and non-repeating excerpts mark the presentation of that pattern.

All sounds were synchronous tone trains containing tones with 42 ms duration (cosine on-and off-ramps of 5 ms). Parameters for these trains were selected based on pilot testing aimed at identifying suitable performance levels with *d′* for the repetition detection task at or near 1.0. The “synchronous” aspect of the trains corresponded to two tones combining to form a multitone complex. The frequencies making up a complex were randomly selected from 500 possible frequencies spaced between 300 and 1,200 Hz on a log scale. Patterns were then made by combining 5 randomly generated complexes consecutively. This made patterns of 210 ms in duration. For each listener, there were three types of patterns. A “Familiar-Fixed” (FF) and “Unfamiliar-Fixed” (UF) pattern were determined at the beginning of the experiment. The seed of Matlab’s random number generator was reset at the start of an experimental session to assure unique fixed patterns for every listener. “Unfamiliar-Unfixed” (UU) patterns were generated randomly throughout the experiment. In relation to previous work, the UF *vs*. UU comparison has been made several times (see intro). The FF *vs*. UF comparison is the novel and relevant comparison for the current study, with the UU condition serving as a control for procedural learning (e.g., learning what “repeat” means, learning the timing of pattern repeats, etc.). In the repetition detection phase, patterns could also be combined to create repeating or non-repeating long duration sound excerpts. In non-repeating excerpts, 20 consecutive patterns (all different) were combined consecutively to make a 4,200 ms excerpt. In repeating excerpts, every other pattern of the 20 consecutive patterns was the same. This procedure created a stimulus with a repeating pattern that was interleaved with other sounds immediately preceding and following it. See Figure 1 for spectrogram depictions of example stimuli.

### Procedure

There were two phases of the experiment. A familiarization phase employed a categorization task intended to familiarize listeners with the FF pattern. On each trial either an FF or a UU pattern was presented. Listeners’ task was to indicate whether the pattern was a “Sound A” type or a “Sound B” type. The FF pattern was assigned to the “Sound A” category, while UU patterns were assigned to the “Sound B” category. Listeners made responses by clicking on-screen buttons corresponding to these labels. There was no response deadline. They received on-screen feedback in the form of “Correct” or “Wrong” text presented for 1.5 s after each response. There were 3 blocks with 15 FF presentations and 15 UU presentations in each block. Order was completely randomized within a block. In between each block, an irrelevant 15 s silent video was presented to give listeners a break from listening and to mitigate fatigue. All videos were neutral valence (e.g., nature-, recreation-, or transportation-related; *cf.* Wisniewski et al., 2019).

The second experimental phase involved a pattern repetition detection task. On half of trials a repeated excerpt was presented. On the other half of trials a non-repeating excerpt was presented. Listeners’ task was to click on a GUI button labeled “no repeats” or “some repeats.” They were instructed to value accuracy over RT, but to respond as soon as they knew the answer. For repeated excerpt trials the repeating pattern could either be an FF pattern, a UF pattern, or a UU pattern fixed within that one excerpt (i.e., the same UU pattern was not fixed across trials). There were also three different types of non-repeating excerpt trials. All patterns were different within each non-repeating excerpt, with a single FF, UF, or UU pattern contained within at a randomly determined position. The position was determined randomly on each trial with equal probability for any position in the excerpt (1–20). This was done to assure that detection of repeats for the FF and UF conditions was not due solely to the recognition of a familiar pattern (*cf.* Agus and Pressnitzer, 2013). Note that this repetition detection task is based on detected repeats of a pattern, not explicit recognition of a pattern from the training phase.

Trials were organized in 12 trial blocks with 2 of each type of trial in each block: 2 repeating FF, 2 repeating UF, 2 repeating UU, 2 non-repeating FF, 2 non-repeating UF, and 2 non-repeating UU. Order within blocks was completely randomized. Feedback of correctness was given at the end of each block in percent correct. There were 10 total blocks for a total of 120 trials, with 40 trials per condition (20 repeating, 20 non-repeating).

### Performance measures

For the familiarization phase, the signal detection *d′* measure was used to determine whether listeners had become familiar with the FF sound. Signal detection *d′* along with median response times were analyzed for the repetition detection phase. To characterize the temporal dynamics of learning, a 10 trial running average (boxcar window) of the hit (“some repeats” responses to repeating excerpts) and false alarm (“some repeats” responses to non-repeating excerpts) rates was taken. The running average window length of 10 trials was chosen based on previous data showing minimal distortion of true *d′* for ~10 trials within the *d′* range observed for the current data (Miller, 1996). These were used to create a running average *d′* using equation 1. Here, H represents the hit rate and F represents the false alarm rate. Where there were hit and false alarm rates of 1 or 0, the rates were adjusted to 1–1/(2n) and 1/(2n) respectively, where n is the number of trials in the window (Macmillan and Creelman, 2005). Median response times were also taken for hits (correct response on repeating trial) across the same 10 trial sliding window.


(1) $$d^{\prime }=z\left(H\right)-z\left(F\right)$$

### Statistics

Linear mixed effects models were used. All models were fit in Matlab’s statistics toolbox using maximum likelihood. First, a linear mixed effects model was fit to *d′* and hit RT data with sound type (FF, UF, and UU), window (centered before fitting), and the sound type x window interaction as fixed effects. Sound type was reference coded to FF. Listener intercept and slopes for sound type and window were entered as random effects. The significance of fixed effects was assessed by comparing the likelihood of this model to those of models where the effect of interest was absent. A value of *p* for each fixed effect was generated by comparing the observed ratio of likelihoods to a χ^2^ distribution with *df* being the difference in number of coefficients for the full and reduced model (Singmann and Kellen, 2019). Any *p*-values less than *ɑ* = 0.05 were deemed significant. Significant effects were followed by post-hoc tests of regression coefficients interpreted with Bonferroni corrections (uncorrected *p*-values reported).

## Results

Listeners learned the categorization task adequately in the familiarization phase. Mean sensitivity (*d'*) to Sound A *vs*. Sound B differences was 1.78 (SD = 1.11), 2.82 (SD = 0.87), and 3.14 (SD = 0.75) in blocks 1–3, respectively. A sign test showed sensitivity in the last block of the familiarization phase to be significantly above zero, *χ*^2^ = 30, *p* < 0.001. Thus, listeners moved on to the repetition detection phase with familiarity for their unique FF sound pattern.

**Figure 2:**
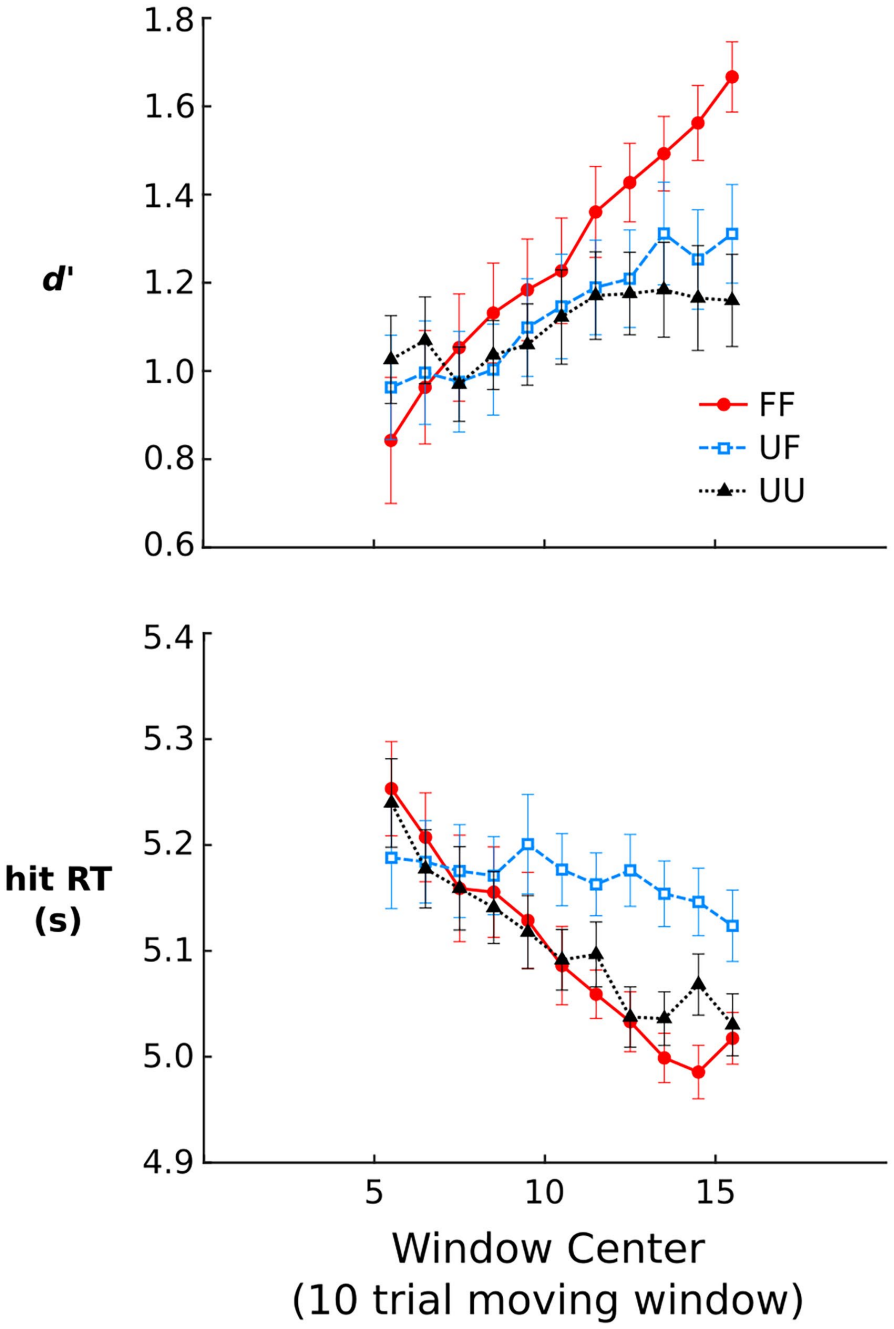
Repetition detection phase data. Signal detection *d*‘ and median RT for hit trials. Signal detection *d*‘ was computed using running average hit and false alarm rates where each rate reflected the contribution of 10 different trials. Median response times (RT) are shown for repeating trials that were correctly detected. All error bars represent within-subject standard errors of the mean (Cousineau, 2005).

Figure 2 shows *d′* and hit RTs over the course of the repetition detection phase. For *d′*, there was a significant effect of window, *χ*^2^ = 23.43, *p* < 0.001, demonstrating increased sensitivity over the course of the repetition detection task. There was also a significant sound type x window interaction, *χ*^2^ = 43.58, *p* < 0.001, demonstrating that the rate of learning was not equal across sound types. Indeed, the model estimated learning slope for FF sounds was significantly steeper than UF, *β* = −0.04, *t* = 4.36, *p* < 0.001, and UU sounds, *β* = −0.06, *t* = 6.57, *p <* 0.001. UF sounds showed no significant difference in learning slope compared to UU sounds, *p* > 0.10. The effect of condition was not significant, *p* > 0.10.

The relatively long length of RTs shown in Figure 2 likely reflects the fact that the task was difficult and participants were instructed to value accuracy over response speed. Nevertheless, RTs on hit trials showed a significant effect of window, *χ*^2^ = 16.42, *p* < 0.001, owing to RTs reducing in latency over the course of the repetition detection task. There was also a significant sound type x window interaction, *χ*^2^ = 27.98, *p* < 0.001, demonstrating differences in the slope of these RT reductions. Model estimated learning slope was significantly steeper for FF sounds, *β* = 0.02, *t* = 5.22, *p <* 0.001, and UU sounds, *β* = 0.01, *t* = 3.98, *p <* 0.001, compared to UF sounds. The slope for FF and UU sounds was not significantly different, *p* = 0.094. The effect of condition was not significant, *p* > 0.10.

## Discussion

The current study was designed to determine whether familiarization with a meaningless sound pattern would facilitate the ability to detect repeated presentations of that pattern among acoustically similar distracters. Familiarity was induced through a categorization task in which a randomly generated pattern of multitone complexes was assigned to one category (FF type) while other randomly generated patterns (UU types) were assigned to another. Though initial sensitivity to FF sounds in a following repetition detection task was comparable to unfamiliar sounds, FF sounds showed a learning advantage. Sensitivity (*d′*) improved at a faster rate and reached a higher level compared to sounds that were repeated throughout the repetition detection task (UF), or that were generated randomly on each trial (UU). Response times decreased over the course of the repetition detection task, but did so at a faster rate for FF and UU sounds compared to UF sounds.

The processes leading to the familiarity effects observed here are not likely to be based in any procedural type of learning. This is because any procedural learning (e.g., developing a concept for what “repeat” means, learning the repetition rate of repeats, etc.) should have been equal across all sound types. It is also unlikely that differences among sound types were related to learning a specific repetition sound quality during the familiarization phase. This is because no consecutive presentations of the FF sound occurred at a rate as fast as that experienced in the repetition detection task (e.g., from trial-to-trial), nor were presentations isochronous during categorization training. The impacts of familiarity are likely perceptual and based in memory for the FF sound itself. This is in line with previous conclusions on learning in UF *vs*. UU sound type comparisons with Gaussian noise samples (Agus and Pressnitzer, 2013). Though in that study all learning did take place within the repetition detection task, those authors showed an increased false alarm rate to trials in which a single familiar pattern was presented. This showed that UF patterns were detectable even without repeats. Together, theses studies suggest that learning in repetition detection tasks reflects the learning of a specific sound pattern.

An advantage of UF over UU sound types has been consistently demonstrated in repetition detection tasks similar to the one used here (Agus et al., 2010; Agus and Pressnitzer, 2013; Andrillon et al., 2015; Viswanathan et al., 2016; Herrmann et al., 2021). However, no such advantage was observable in this study. Rather, the only difference between UF and UU sounds appeared as a disadvantage for the former in learning as measured by RT. This failure to replicate might be related to the interleaving with distracter sounds (i.e., more informational masking), the lack of immediate feedback, and/or the accompanying FT sound type. Further speculation would require an alternative experimental design. Nevertheless, the FF *vs*. UF comparisons shed light on the impact that familiarity with meaningless sound patterns can have on repetition detection performance and learning. That differences between FF and UF sounds appeared over the course of learning is consistent with a recurring idea in perceptual learning theory that the benefits of familiarization with a stimulus lie in the impact on learning potential (e.g., accelerated learning rates or capacity to learn; Gibson, 1969; Mercado III, 2008; Goldstone et al., 2010). Prior research on this hypothesis has mostly employed familiar speech sounds (*cf.* Wisniewski et al., 2014), known environmental objects (for review, see Fine and Jacobs, 2002), or preexisting individual differences in perceptual acuity (e.g., Orduña et al., 2005). Familiarity with these methods comes from an unknown learning history with learned perceptual and social biases (e.g., towards one spoken accent or another; Gluszek and Dovidio, 2010). In contrast, this study produced familiarity effects which are unlikely to have any contribution from preexisting biases of listeners. FF versus UF comparisons in tasks like the one use here will be of use to further the study of familiarity effects in perceptual learning without these confounding biases.

Future research studies are needed to tease out the roles of specific learning processes in the effect of familiarity on learning rates observed here. One possibility is that a filter is developed during the familiarization phase based on predictable sound patterns (Heilbron and Chait, 2018). This could then transfer over to the repetition detection task (e.g., Amitay et al., 2014). A filter such as this could operate to separate previously experienced patterns (signals) from novel patterns (noise). That filter could be accomplished through long-term plasticity in the auditory processing hierarchy (e.g., Wisniewski et al., 2017; Irvine, 2018) or be dependent on transient shifts in attention (Carlin and Elhilali, 2015). Another possibility is that representations of initially “meaningless” sounds build to meaning over the course of the familiarization phase. For instance, an FF sound could end up being represented by the Sound A label/category. When performing the repetition detection task, listeners may be able to use this representation at a higher-level of the auditory hierarchy to enhance learning (Ahissar et al., 2009). This study has laid out a paradigm in which these possibilities can be further investigated.

## Data availability statement

The raw data supporting the conclusions of this article will be made available by the author, without undue reservation.

## Ethics statement

The studies involving human participants were reviewed and approved by Kansas State University Institutional Review Board. The patients/participants provided their written informed consent to participate in this study.

## Author contributions

The author confirms being the sole contributor of this work and has approved it for publication.

## Funding

This research was supported by the Cognitive and Neurobiological Approaches to Plasticity (CNAP) Center of Biomedical Research Excellence (COBRE) of the National Institutes of Health under Grant No. P20GM113109. Publication of this article was funded in part by the Kansas State University Open Access Publishing Fund.

## Conflict of interest

The author declares that the research was conducted in the absence of any commercial or financial relationships that could be construed as a potential conflict of interest.

## Publisher’s note

All claims expressed in this article are solely those of the authors and do not necessarily represent those of their affiliated organizations, or those of the publisher, the editors and the reviewers. Any product that may be evaluated in this article, or claim that may be made by its manufacturer, is not guaranteed or endorsed by the publisher.
